# Diatom–Bacteria Interactions in the Marine Environment: Complexity, Heterogeneity, and Potential for Biotechnological Applications

**DOI:** 10.3390/microorganisms11122967

**Published:** 2023-12-12

**Authors:** Federica Di Costanzo, Valeria Di Dato, Giovanna Romano

**Affiliations:** Stazione Zoologica Anton Dohrn Napoli, Ecosustainable Marine Biotechnology Department, Via Ammiraglio Ferdinando Acton 55, 80133 Napoli, Italy; federica.dicostanzo@szn.it (F.D.C.); giovanna.romano@szn.it (G.R.)

**Keywords:** marine diatoms, marine bacteria, microbial interactions, microbiomes, biotechnological applications

## Abstract

Diatom–bacteria interactions evolved during more than 200 million years of coexistence in the same environment. In this time frame, they established complex and heterogeneous cohorts and consortia, creating networks of multiple cell-to-cell mutualistic or antagonistic interactions for nutrient exchanges, communication, and defence. The most diffused type of interaction between diatoms and bacteria is based on a win-win relationship in which bacteria benefit from the organic matter and nutrients released by diatoms, while these last rely on bacteria for the supply of nutrients they are not able to produce, such as vitamins and nitrogen. Despite the importance of diatom–bacteria interactions in the evolutionary history of diatoms, especially in structuring the marine food web and controlling algal blooms, the molecular mechanisms underlying them remain poorly studied. This review aims to present a comprehensive report on diatom–bacteria interactions, illustrating the different interplays described until now and the chemical cues involved in the communication and exchange between the two groups of organisms. We also discuss the potential biotechnological applications of molecules and processes involved in those fascinating marine microbial networks and provide information on novel approaches to unveiling the molecular mechanisms underlying diatom–bacteria interactions.

## 1. Introduction

Diatoms are among the most successful phytoplanktonic organisms, thriving in all aquatic environments [1], where they have to cope with competitors, pathogens, and grazers [2,3]. To defend themselves, they have developed different strategies, including the production of secondary metabolites affecting the reproduction, fitness, and viability of grazers, competing microalgal species, and bacteria. The interactions with bacteria living in their surroundings are particularly crucial to success in most environments [4]. Bacteria assimilate nutrients from the sea and sequester minerals with high efficiency, competing with microalgae, including diatoms, for resources while establishing positive collaborations with some other species based on the exchange of nutrients fundamental for both [5]. The so-called “microbial loop”, described for the first time by Azam et al. [5], plays a pivotal role in the flux of energy and nutrients in the marine environment. In this loop, bacteria contribute by assimilating mineral nutrients and recycling organic matter deriving from dying phytoplankton organisms, among which diatoms, thus reintroducing them into the food web by transferring to protozoa, heterotrophic microalgae, and zooplankton, up to higher trophic levels.

The interactions between diatoms and bacteria are fundamental in shaping population dynamics, are not only related to nutrient exchange, and are often complex and heterogeneous, spanning from mutualistic to antagonistic [6,7,8]. However, despite the importance of such interactions in the evolutionary history of diatoms, in structuring the marine food web, and in controlling algal bloom termination, the molecular mechanisms and factors underlying this crosstalk remain poorly studied [9]. The existence of an inter-kingdom mechanism of signalling between bacteria and diatoms has been confirmed only in the last decade [10]. In this interplay, bacteria communicate through the production and release of quorum sensing (QS) molecules, while diatoms produce pheromones possessing functionalities similar to bacterial QS molecules.

Recent studies based on the analysis of the entire microbiome associated with diatoms provided a comprehensive perspective on diatom–bacteria relationships [6], also describing the role of the phycosphere in these interactions [7,8]. Despite a growing interest in this topic [4,8,11], few studies integrate, from a global perspective, the different types of interactions and factors involved. Hence, this review aims to provide a comprehensive report illustrating the different interplays occurring between these two groups of organisms in the marine environment, the chemical mediators involved, and describing some innovative approaches utilised to understand those interesting and fascinating marine microbial networks. A general view and a good comprehension of the mechanisms and molecules at the base of diatom–bacteria interplay are essential to allow the exploitation of the potential natural or synthetic consortia for applications in the marine biotechnology field, such as for bioremediation purposes or for the sustainable production of valuable compounds, eventually leading to the development of new feed and food, cosmetic formulations, or new drugs.

## 2. Diatom–Bacteria Niches of Interactions

The ocean is a heterogeneous environment in which nutrients are not uniformly distributed but are pumped into the water column by biotic and abiotic sources and spread by turbulence and currents, creating local nutrient patches [12]. Consequently, bacteria, microalgae, and other members of the marine plankton are aggregated in “hot spots” [12], in which nutrient exchanges, communication, and defence are facilitated [13,14]. In those spots, interactions between diatoms and bacteria may occur both through chemo-attraction and attachment to the cell surface, which are mediated by QS in the case of motile bacteria [15], or more rarely by random encounters and collisions due to water movements in the case of non-motile bacteria [4,16,17]. These microenvironments have different features, and the most common are marine snow in open waters [18] or biofilms on the surface of different substrates in the photic zones [13,19]. A relevant role in establishing interactions among diatoms and bacteria is played by the phycosphere [8,20].

### 2.1. Phycosphere

**Figure 1:**
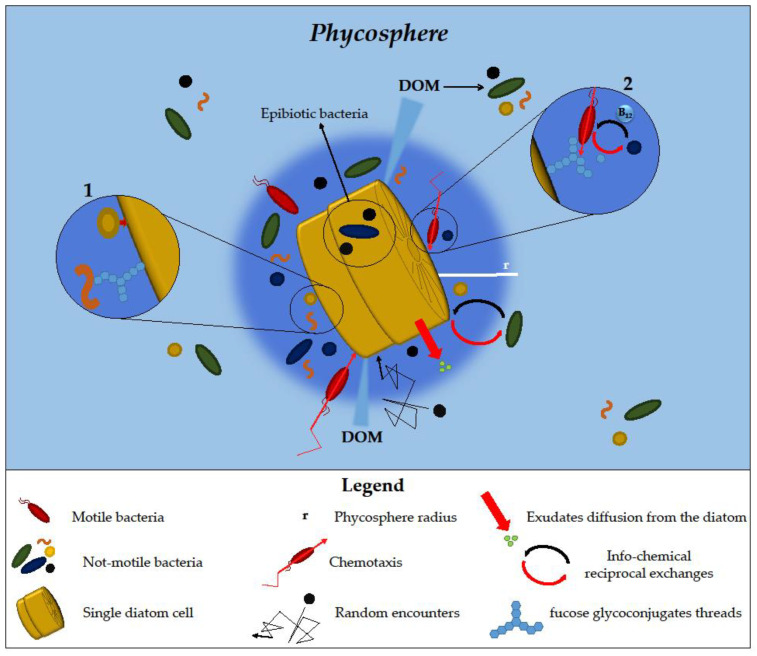
Phycosphere features and diatom–bacteria interactions occur in it. The phycosphere is represented as a dark blue gradient. The different types of interactions and types of contact are illustrated in the zoom-in circles (details in the text). Zoomed-in circle n° 1: bacteria attached to diatom cells by direct contact or to fucose glycoconjugate threads. Zoomed-in circle n° 2: bacteria unable to directly bind glycoconjugates benefit from their degradation by other bacteria possessing polysaccharide-degrading enzymes.

The phycosphere is the physical space surrounding the diatom’s cell surface, in which nutrients and exudates are mostly concentrated and the exchanges between diatoms and bacteria occur to a greater extent [8,20] (Figure 1).

In general, phycosphere size depends on the size of phytoplankton cells, growth, and exudation rates that set different phytoplankton-phycosphere radii [8]. Since the turbulence is not able to influence water layers at a smaller scale, the transport of exudates from diatoms occurs mainly by diffusion [4]. However, when the phycosphere and cell radius are over a certain threshold size, mild to moderate turbulence can influence the diffusion rate and the phycosphere size itself by stirring and stretching its shape and mixing the exudate gradient [8]. Due to the diversified interactions occurring and the variability of its composition and structure, the phycosphere can be considered a very dynamic environment [21]. This milieu offers several advantages to the bacterial community associated with microalgae by providing them with easier access to organic matter released compared to those free living in the water column [15]. This may lead to an increased local concentration of bacteria in the phycosphere, as it was shown that richness in particulate organic matter may increase bacterial species diversity and abundance [22] (Figure 1).

The stability of the phycosphere and the interactions occurring in it are influenced by the chemical composition of exudates released by the diatoms. Indeed, a greater loss of small molecules compared to large ones generally occurs since the latter diffuse at a lower rate, which results in an increase in their residence time in the phycosphere, ultimately influencing its size and stability [8]. This may generate a chemotactic gradient, attracting coloniser bacteria that often form consortia with commensal or mutualistic relationships, degrading diatom-released compounds [23]. On the other side, bacteria may also influence the chemical composition of the phycosphere, with some differences related to the diversity of metabolism in different species [4,8,16]. In particular, it was shown that when *Phaeodactylum tricornutum* CCAP 1055/15 was co-cultured in a dual system with the marine bacterium *Pseudoalteromonas haloplanktis* TAC125, the interactions occurring in this simplified model significantly modified the composition of the exo-environment, as evaluated by the monosaccharide profiles of extracellular polymeric substances (EPS) [24].

Confocal laser scanning microscopy (CLSM) imaging showed that some bacteria stay in the phycosphere by either binding directly to the diatom cell surface or attaching to fucose glycoconjugate threads extending from diatom cells, as observed for bacteria associated with *Thalassiosira rotula* [25]. On the contrary, other bacteria associated with the same diatom are not able to directly attach to the diatom cell surface needing to interact with glycoconjugate-binding bacteria in order to colonise diatom cells. Bacteria possessing polysaccharides degrading enzymes can be attracted by threads and tufts of diatoms that can be used as carbon sources, favouring the establishment of cooperative association with other bacteria, as happens for *Gramela forsetii* that degrade the fucose glycoconjugate, making them available for *Planktotalea frisia* that in return produce vitamin B_12_, for which *G. forsetii* is auxotroph (Figure 1, Zoomed-in circles n° 1 and 2).

Among bacteria interacting with diatoms, the epibiotic ones, that are attached to diatom cell surfaces, often have been found between and underneath a specific region of the diatom frustule, the cingulum, where they are more protected against several stressors while taking advantage of organic matter released by diatoms in the surroundings of this structure [26] (Figure 1).

For motile bacteria, the phycosphere colonisation can involve a QS mechanism that acts on the biphasic “swim or stick” behaviour possessed by many bacterial genera, influencing their passage to a different lifestyle [15]. Indeed, being attracted in the phycosphere towards the nutrients, once there, they become resident by losing the flagella and forming biofilms. Fei et al. [15], while studying the phycosphere of *A. glacialis,* identified in three symbiotic bacteria strains, isolated from its associated microbiome community, five chemotaxis genes (organised in one cluster), plus other genes involved in pilus formation, exopolysaccharide production, and biofilm formation. These genes were differentially present in the associated bacteria species, and the only two bacteria capable of colonising the diatom were the ones with the distinctive feature of producing the QS effectors 3-oxo-C16-HSL, 3-oxo-C10-HSL, and 3-oxo-C_16:1_-HSL. These last are indeed necessary to reduce bacteria motility and stimulate their attachment to diatom-produced transparent exopolymeric particles (TEPs), with 3-oxo-C_16:1_-HSL being the strongest inhibitor of bacteria motility.

Metagenomics and metaproteomics analysis of bacteria occurring during phytoplankton blooms in the North Sea revealed a “tight adherence (*tad*)” gene cluster codifying for the formation of pili, allowing the attachment on the diatom surfaces and the colonisation of the phycosphere in a subset of bacteria belonging to the order Rhodobacterales [27]. Those bacteria relied on *tad* in combination with QS, motility, and chemotaxis to colonise the phycosphere.

Diatoms also contribute to the selection of the bacteria species entering the phycosphere. This is the case of *A. glacialis*, which releases rosmarinic acid (RA) and azelaic acid (AA), which are able to selectively promote the motility and growth of specific bacteria, operating as a selection agent [28]. Transcriptomics, metabolomics, and co-expression network analysis revealed that *Phycobacter* was able to assimilate and catabolize AA using it as a carbon source, while *Alteromonas macleodii* showed the activation of a stress response mechanism based on the activation of cytoplasmic efflux and the downregulation of ribosome and protein synthesis pathways [29], confirming that AA is able to mediate microalgae-microbiome interactions.

The phycosphere displays some similarities with the plant rhizosphere, the interface between plant roots and soil [30]. As already reviewed by Seymour et al. [8], in both microenvironments, interactions with microorganisms are established and driven by the chemotactic perception of plant-released organic compounds, and some microbial taxa can be detected at both types of interfaces, such as *Rhizobium* and *Sphingomonas*. Similarly to the rhizosphere environments, bacteria–phytoplankton interactions in the phycosphere influence growth, health status, and response to biotic and abiotic stresses, but also contribute to nutrients biogeochemical cycles [30]. Some chemical signalling molecules are common between the two microenvironments, such as oxylipins [31] that play a role in several physiological processes in plants, such as growth and development, and are involved in the defence against pathogens, showing antimicrobial activities against bacteria and fungi [32]. In addition, jasmonic acid (JA), a molecule deriving from a path parallel to the oxylipins one, plays a central role in plant defence against necrotrophic pathogens and, on the other hand, is also activated when plants encounter beneficial bacteria that trigger plant-induced systemic resistance [33]. This compound also influences rhizosphere microbial composition by altering roots’ exudate profile.

Diatoms also produce and release oxylipins, some of which are involved in the defence against algicidal bacteria [34,35] (further details in Section 3.3.2). While JA has not been detected in diatoms and generally in phytoplankton, prostaglandins (PGs), structurally similar to JA [31], have been recently discovered in these microalgae [36,37]. PGs roles have not yet been clarified in diatoms, but their release by *T. rotula* cells in the environment increases during stationary and senescent phases of growth, allowing us to hypothesise for these molecules a possible role in the interaction of diatoms with bacteria [37].

### 2.2. Marine Snow

**Figure 2:**
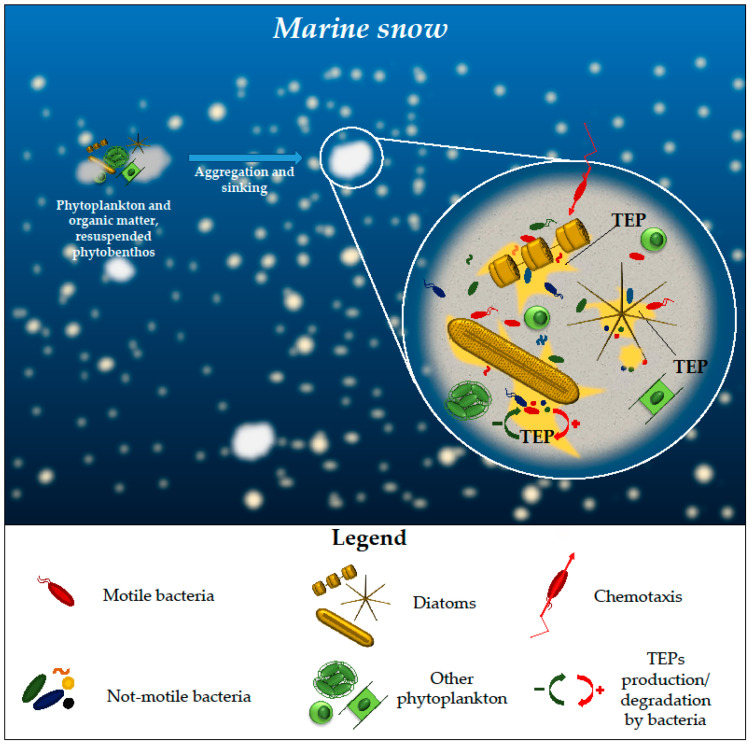
Marine snow formation and composition. The background gradient from light to dark blue indicates the sinking from the upper to the lower water layers occurring when diatoms, followed by other phytoplankton algae, aggregate with organic matter at the end of blooms. A zoom on a single particle highlights the presence of bacteria and of different phytoplankton groups. Bacteria can reach these aggregates by chemotaxis. Transparent exopolymeric particles (TEPs) released by diatoms and bacteria are responsible for the cohesion of the particles. Bacteria are also able to degrade TEPs produced by diatoms, influencing the aggregation process.

Diatom aggregation is one of the main driving forces in marine snow formation, occurring commonly at the end of diatom blooms due to the collision and aggregation of the cells with organic matter and, to a lesser degree, from the resuspension of benthic biofilms [38]. Marine snow patches, being rich in nutrients and widely distributed, transport organic matter from the upper to the lower layers [39] (Figure 2).

The quality and quantity of organic matter transported to the deep ocean by sinking particles are greatly influenced by bacterial succession [40]. In a network analysis conducted on a 3.5-year sampling dataset, it emerged that Flavobacteriia and some Gammaproteobacteria, among which *Pseudoalteromonas* and *Alteromonas* genera, use chemotactic motility to detect and rapidly reach diatom aggregates, contributing with other bacterial species to their degradation in the dark ocean. In particular, *Pseudoalteromonas*, which may be among the first colonisers, exerts the key initial steps of cell degradation, making nitrogen and carbon compounds accessible to other bacteria. Then *Alteromonas* starts to uptake nitrogen compounds deriving from aggregate degradation while colonising them only at a later time.

One of the main agents training the aggregation are TEPs, principally released by diatoms [38] but also by bacteria and by phytoplankton in general [41] (Figure 2). TEPs are acid-rich polysaccharides that can be formed both abiotically and biotically through the condensation or exudation of extracellular polymeric substances (EPS) [42]. The production of diatom TEP precursors is species-specific. Different diatom species can produce them in low or high quantities, while others do not produce them at all [18]. For high TEPs producing diatoms, it seems that the aggregation of particles is part of their life cycle, contributing to their sinking at the end of the blooms. Polyunsaturated aldehydes (PUAs), released by diatoms in high quantities at the terminal phase of the blooms or through wound-activated mechanisms [43], have a dose-dependent effect on TEP formation [44] and on the community composition of the bacteria associated with sinking particles [45]. Indeed, while low concentrations of PUAs are able to stimulate organic matter remineralization and metabolic activity of bacteria associated with them [45], higher concentrations of PUAs are able to increase TEP aggregate size [44], cause a shift in the community composition, and overall reduce bacterial growth [45].

On the other side, marine bacteria are also able to produce TEPs, which increases cell adhesiveness, favouring aggregation, or degrade TEPs, obtaining organic matter [18,46,47]. As an example, while bacteria enhance *T. rotula* aggregation, they prevent it in *Skeletonema costatum* through consumption of the organic carbon released by the diatom, which consequently affects TEP formation [47]. Moreover, while axenic *Thalassiosira weissflogii* does not aggregate, the reintroduction of its strictly associated bacteria increases cell aggregation [18]. An active photosynthesis process, being responsible for the production of precursors, is an important factor determining TEP formation, as demonstrated by the fact that photosynthetically inactive diatoms do not aggregate independently from the presence of bacteria.

### 2.3. Biofilms

**Figure 3:**
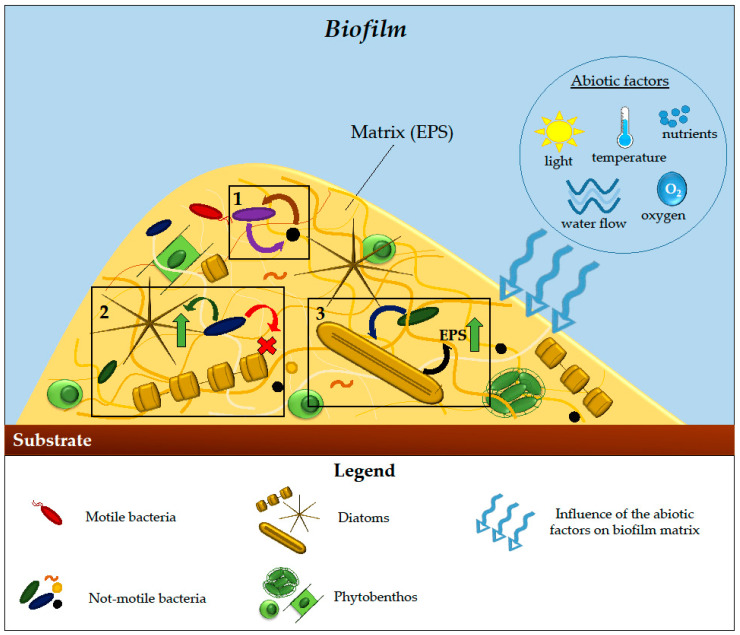
Biofilm structure and composition. The figure shows interactions that take place in biofilms. Curved arrows indicate the direction of the influence. The different types are grouped in black boxes: (1) bacteria influencing the behaviour of other bacteria living in the biofilm; (2) positive (green arrow) or negative (red cross) effect of bacteria on the dominance of different diatom species; (3) stimulatory effect (green arrow) on diatom extracellular polymeric substances (EPS) production. Abiotic factors, such as light, water flow, temperature, oxygen, and nutrient composition, are able to influence biofilm structure and stability.

Biofilms represent one of the most widespread modes of aggregation among microorganisms, whose interaction actively regulates organic matter cycles and energy fluxes [13,19]. They represent a hub for microorganisms’ aggregation where metabolic cooperation, cell-to-cell communication, genetic exchanges, protection against grazers, pathogens, toxins, and environmental stressors are favoured [13]. Diatoms are among the first colonisers of organic and inorganic surfaces in the marine environment, representing an important component of the biofouling community [48]. Pennate diatoms, having predominantly a benthic lifestyle, often dominate biofilms. Among them, the most widespread species belong to the genera *Navicula*, *Amphora*, *Nitzschia*, *Pleurosigma*, and *Thalassionema* [49]. Biofilms are composed of a matrix of EPS, mainly produced by bacteria and diatoms, that can be colonised by autotroph and heterotroph organisms, among which other bacteria, fungi, protozoa, and algae [50,51], all establishing connections among them and with the substrates [19] (Figure 3). EPS comprise different organic macromolecules, mainly polysaccharides but also glycoproteins and other organic polymers [50,52] and can be bound to the cells or released in the medium [19]. The EPS have a key function in mediating the initial attachment of cells to different substrata and provide protection against environmental stress [53].

The EPS matrix and its modifications occurring during diatom–bacteria interaction are involved in the biogeochemical cycle of carbon, nitrogen, and sulphur, and they are also relevant for a wide range of metal cations [42]. The binding of metals to EPS is considered fundamental for their vertical transport in the water column and their final accumulation into the sediments at the depth where they are transformed and finally re-enter the food chain [54]. Among the different diatom species, the polysaccharides present in the released EPS show high diversification in their molecule structures and branching [55]. The most frequently secreted ones are composed of galactose and glucose, but often also of xylose, rhamnose, mannose, and fucose [56,57,58,59] that can be used as carbon sources by bacteria [59,60]. These are able to influence the EPS composition of the biofilms as they secrete EPS themselves and may influence the formation and changes in the matrix composition through the release of chemicals, especially QS molecules [61]. A study supporting this evidence was conducted in the laboratory on the diatom *P. tricornutum* that was co-cultivated with *Escherichia coli* or with the bacterial spent medium, demonstrating that soluble substances produced by the bacteria can influence diatom growth and EPS secretion [62]. In another example, biofilm formation by the benthic diatom *Cylindrotheca* sp. was shown to be induced by exposure to N-Acyl homoserine lactones (AHLs) produced by bacteria, which stimulated EPS secretion and chlorophyll-a content, consequently enhancing the diatom biomass [63].

Bacteria can also influence the species balance inside the biofilms (Figure 3). An example is the change in species prevalence when a mixed axenic co-culture of the three benthic diatoms *Navicula phyllepta*, *Seminavis robusta*, and *C. closterium* is added with a freshly prepared bacterial inoculum from marine sediments [64]. While in the axenic co-culture *C. closterium* was the dominant species, after the addition of bacteria isolated from intertidal surface mud, its growth and that of *N. phyllepta* were impaired, while *S. robusta* became the dominant species. Interestingly, each of those species cultivated individually with the same bacterial inoculum developed a specific bacterial community that became a combination of the three when the diatom species were cultivated together. In addition, the biomass growth rates of the diatom monocultures were negatively influenced by the added bacteria, while the co-cultures were not impacted, suggesting that the contemporary presence of different diatom and bacterial species positively influences the productivity rate.

Apart from biotic factors, biofilm community composition, structure, and stability can also be influenced by the combination of abiotic factors, such as the substrate’s composition, its physical and chemical properties, temperature, nutrient availability, light, oxygen, and water flow velocity [65] (Figure 3). For example, it has been observed that low light intensity impaired biofilm development, as light is fundamental for diatom growth and EPS production [66]. The same study reported that high water flow velocity delayed biofilm formation since turbulence made it difficult for microalgae to settle on the substrate. In these unstable biofilms, the community was dominated by opportunistic and motile species, while sessile diatoms and bacteria dominated those unperturbed.

## 3. Diatom–Bacteria Types of Interactions

Diatom–bacteria interactions may occur through the exchange of metabolites, from small volatiles to complex molecules [8,67]. Such compounds are produced and released by both organisms to communicate, protect themselves, or impair the survival of their counterparts, ultimately contributing to the functioning of the ecosystem [68].

One example of metabolites produced by diatoms serving as defence strategy and also influencing the ecosystem equilibrium is the neurotoxin domoic acid (DA), produced in higher quantities during the stationary growth phase by some diatoms of the genera *Pseudo-nitzschia* and *Nitzschia* [69]. Silicate, phosphate, and iron limitation, as well as the change in nitrogen sources, seem to influence DA production [70,71,72,73]. There is also evidence that biotic factors, such as the presence of non-autochthonous bacteria able to utilise DA as a source of organic matter [11,26,74,75,76,77], may affect DA biosynthesis. However, the molecular mechanism underlying the influence of these bacteria on DA production has not yet been clarified [78]. It has been observed that the DA-producing *Pseudo-nitzschia multiseries* harbours a less abundant and diversified bacterial community than the not-toxic *Pseudo-nitzschia longissima*, although these diatoms belong to the same genus [79]. This finding suggests that the bacterial community is influenced by DA and by other strain-specific cues released by DA-producing diatoms.

Interactions between diatoms and bacteria may involve different mechanisms. Here we focus on mutualistic, facilitative, and antagonistic interactions. The first is based on the exchange of nutrients and the release of growth factors that is beneficial for both partners; the second is based on the benefit for only one of the organisms involved in the relation without damage for the other [4,8]; the third implies the inhibition of growth or the release of algicidal compounds by bacteria [7,80], which leads to the death of co-occurring diatoms.

Diatom–bacteria interactions may be affected by several factors, such as the phase of the growth of both organisms involved and the cultivation conditions. A study of pairwise interactions between 8 diatom species and 16 bacterial strains highlighted that these relationships were strain-specific, with regards to bacteria, and may depend on the growth phase of the diatom. Overall, no universally positive or negative effects on diatom growth have been observed [81]. In particular, the bacterium *Mameliella* sp. was able to inhibit the growth of *Chaetoceros socialis* while improving the growth of *Coscinodiscus wailesii.* In addition, *Coscinodiscus radiatus* was affected in opposite ways by different bacteria, as all Gammaproteobacteria inhibited the growth of the diatom during its early growth phase, while all *Roseobacteraceae* stimulated its growth in the late growth phase. On the other hand, *Maribacter* sp. was the only bacterium promoting the growth of all the diatoms tested when they were in the late growth phase.

Nutrient availability can also influence bacteria–diatom population dynamics, as demonstrated by the capability of *A. macleodii* to have positive, negative, or neutral interactions with the diatom *P. tricornutum*, depending on nitrogen availability [82]. Specifically, *A. macleodii* establishes commensal relationships with *P. tricornutum* during its exponential growth, with no impact on diatom growth, when nitrogen is abundant, benefiting from the dissolved organic carbon (DOC) released by this diatom species. Later, during the stationary phase, both diatoms and bacteria continue to increase their cell numbers, suggesting the occurrence of a cooperative interaction with the bacteria providing the nitrate to the diatom for their growth, while diatoms provide the organic matter to bacteria. Conversely, the addition of DOC to the media at the diatom’s early growth phase, before nitrate becomes depleted, triggers *A. macleodii* proliferation and reduces *P. tricornutum* growth, suggesting that in the presence of sufficient DOC, the bacterium competes with the diatom for nitrogen uptake.

### 3.1. Mutualistic Interactions

A typical mutualistic relationship is based on the exchange of cobalamin (vitamin B_12_), for which many diatoms, as well as many other eukaryotic algae, are auxotrophs [83,84]. A simplified list containing examples of mutualistic interactions is reported in Table 1.

In exchange for organic carbon or nitrogen, selected bacteria and archaea establish symbiosis with diatoms by proving vitamin B_12_ that is produced de novo or through modifications of pseudo-cobalamin and other closely related compounds [97]. However, this system represents an active relationship and not a mere passive exchange of nutrients [85,86]. Indeed, to create a specific association with the bacterium *Ruegeria pomeroyi*, which supplies diatoms with vitamin B_12_, *Thalassiosira pseudonana* releases 2,3-dihydroxypropane-1-sulfonate (DHPS). When in co-culture with *T. pseudonana*, *R. pomeroyi* shows an up-regulation of the genes involved in DHPS catabolism, while the diatom upregulates genes involved in the release of organic compounds, supporting bacterial growth. In a coastal environment in which iron and vitamin B_12_ limitations were found, the main vitamin B_12_ producer in plankton communities was found to be the Gammaproteobacteria *Oceanospirillaceae* ASP10-02a [87]. This bacterium showed high expression of genes related to organic matter acquisition and cell surface attachment, thus entertaining a mutualistic relationship with phytoplankton, among which diatoms, to fuel vitamin B_12_ production. Moreover, *Pseudo-nitzschia subcurvata* exuded organic matter that is used by *Sulfitobacter* sp. SA1 as a source of nutrients, while the bacterium provides biotin, vitamin B_12_, and thiamine that support diatom growth in vitamin-limiting conditions [88]. SA1 also possesses a catalase that, similarly to what was previously observed in another diatom-*Sulfitobacter* association, can further improve *P. subcurvata* growth, supporting it in detoxification processes.

Similarly, diatoms unable to fix atmospheric N_2_ establish symbiosis with nitrogen-fixing cyanobacteria, giving in return amino acids and organic carbon [8,89]. N_2_-fixing cyanobacteria can be both obligate and facultative. As an example, *Richelia intracellularis* is an obligate symbiont adapted to live inside the *Rhizosolenia* and *Hemiaulus* genera frustules and is also transmitted to the host’s next generation [90]. Conversely, *Calothrix rhizosoleniae* is a facultative symbiont living outside the frustule of *Chaetoceros* spp. [91].

Certain bacteria use monomethyl amine (MMA), ubiquitously present in the ocean, as sources of organic carbon, energy, and nitrogen (or the sole nitrogen source) [80]. For example, the strain KarMa of the *Rhodobacteraceae Donghicola* sp. retrieves nitrogen in the form of ammonium from MMA degradation, providing it to *P. tricornutum*, thus sustaining its growth under photoautotrophic conditions. This interaction has a mutualistic character since KarMa growth, in turn, is supported by diatom-released organic carbon. This cross-feeding is widespread, since it was also observed when KarMa was co-cultured with the other two diatoms, i.e., *Amphora coffeaeformis* and *T. pseudonana* [92]. In addition, other *Rhodobacteraceae* strains were able to degrade MMA and establish interactions with diatoms. Similarly to MMA, methylamines (MAs), i.e., di- and trimethylamines, and glycine betaine (GBT) can be degraded by *Rhodobacteraceae* to obtain carbon, nitrogen, and energy. GBT is a quaternary methylated amine osmolyte that can be excreted and re-uptaken by diatoms following environmental salinity fluctuations. However, diatoms are not able to reuse the nitrogen from GBT, thus taking advantage of the associated *Rhodobacteraceae* to maintain a short nitrogen cycle in the phycosphere.

Some hormones, such as indole-3-acetic acid (IAA), may also represent the bridge between bacteria and diatoms to develop mutualistic relationships [93]. Among the *Sulfitobacter* genus, the SA11 strain synthesises IAA using both diatom-secreted and free tryptophan present in the medium as a signal molecule for nutrient exchange with *P. multiseries*. IAA promotes diatom cell division and photosynthetic activity that probably fuel bacteria with organic carbon and organosulfur molecules such as taurine and dimethyl sulfoniopropionate (DMSP). Bacteria, on their side, excrete ammonium useful for diatom growth. DMSP is an osmolyte that also acts as a chemoattractant for bacteria, produced in high quantities by dinoflagellates and low quantities by diatoms [94]. Then bacterial DMSP catabolism provides carbon, sulphur, and the volatile dimethylsulfide (DMS), the main source of natural sulphur in the atmosphere [95]. Finally, it has been observed that the bacterium *Stappia* sp. K01 is able to stimulate *P. tricornutum* growth and pigment production, benefiting from diatom-released fatty acids (FAs) as a nutrient source [96]. Indeed, several diatom-growth-related genes, such as those involved in DNA replication and cell division, are up-regulated when the bacterium is present with respect to the axenic cultures.

### 3.2. Facilitative Interactions

The beneficial effect of bacteria on diatom health and growth has been shown in many studies to occur through different mechanisms, always involving metabolite exchange [67,98,99,100,101,102]. Less is known about possible advantages for the bacterial community growing with diatoms, besides the already-ascertained advantage of benefiting from organic matter released by co-occurring diatoms.

A simplified list of examples of facilitative interactions between diatoms and bacteria is reported in Table 2.

Bacteria are able to influence the diatom metabolic profile by stimulating diatom cells towards the synthesis of amino acids and secondary metabolites. In a study conducted by co-culturing *T. pseudonana* with the bacterium *Dinoroseobacter shibae*, separated by a membrane that allowed only the exchange of chemical signals without any physical contact, diatom abundance was higher in comparison with the axenic algae [67]. Metabolic activity was also increased, especially with regards to the concentration of some intracellular amino acids and their derivatives, while the general health status did not change.

A recent study demonstrated the establishment of a diatom–bacteria metabolic interaction based on the production of the osmoregulatory compound ectoine. This osmolyte is mainly produced by bacteria and, to a lesser extent, by diatoms, allowing the adaptation of cells to salinity changes [101]. When *T. weissflogii* and its microbial community were cultivated under saline stress conditions, an increased content of intracellular ectoine in the algae cells was observed compared to those growing at a normal salinity concentration. This increase was higher in xenic cultures compared to axenic ones, while the increase in other osmolytes, such as homarine and GBT, was higher at high salinities independently from the presence of bacteria. These results led the authors to hypothesise that *T. weissflogii* internalises ectoine released in the media by the bacteria present in the culture to counteract osmotic stress. Similarly, xenic cultures of *T. rotula* producing ectoine were able to survive to salinity concentrations at which the axenic counterparts were not [103]. The authors also showed an increased abundance of ectoine-producing bacteria species when the ectoine levels in the media were high. The transcripts codifying for the enzymes Ect-A and Ect-B, involved in ectoine biosynthesis, have been identified in the transcriptome of this diatom. By amplifying these gene sequences on the *T. rotula* axenic gDNA, the EctB gene was found to be present in the diatom genome rather than the bacterial one, while Ect-A was present in both (Di Dato, personal communication).

The bacterium *Bacillus thuringiensis*, following sporulation and mother cell lysis, releases compounds among which two diketopiperazines (DKPs), which are able to stimulate the growth of *P. tricornutum* as well as its content in neutral lipid [98]. Similarly, a high number of bacteria cells in the cultures stimulated the growth rates and biomass accumulation of the benthic diatoms *Halamphora coffeaeformis* and *Entomoneis paludosa* but lowered the diatom cellular amount of proteins, lipids, and extracellular carbon in the form of EPS [99]. The lower concentration of this last in the xenic cultures was also due to its significant use as a carbon source by the bacteria.

Interestingly, the association with some bacterial species can be very advantageous for diatoms maintenance of genetic diversity and cell size recovery [102]. Indeed, some bacteria increase the diatom sexual reproduction, by which they restore the original size while also reshuffling the genetic material. *Polaribacter* sp. and *Cellulophaga* sp. are examples of bacteria stimulating sexual reproduction in the diatom *Odontella* sp. that had a higher formation rate of sexual cells and auxospores when inoculated with them. *Odontella* was able to produce sexual cells and auxospores when grown in optimal nutrient, light, and temperature condition, but the rate of formation was half or ⅓ compared to the ones in the presence of *Polaribacter* and *Cellulophaga*. None of the other bacteria genera present in the same *Odontella* natural assemblage, i.e., *Vibrio* sp., *Pseudoalteromonas* sp., *Sulfitobacter* sp., and *Planococcus* sp., were able to stimulate sexual cell production.

Protection from viral infections is another positive effect that bacteria offer to diatoms. When *Chaetoceros tenuissimum* was grown in axenic conditions, the infection by the RNA virus CtenRNAV provoked a complete lyse of the diatom cells [100]. Instead, when grown in co-culture with the bacterial genera *Nautella* sp., *Sulfitobacter* sp., and *Polaribacter* sp., the diatom cells survived the viral infection and regrew.

### 3.3. Antagonistic Interactions

#### 3.3.1. Inhibitory Effects of Bacteria on Diatoms

Not always the interactions between diatoms and bacteria are beneficial for one or both of them [4]. Table 3 reports a simplified list of the inhibitory effects exerted by bacteria on diatoms.

An example is given by the Flavobacter *Croceibacter atlanticus*, commonly associated with *Pseudo-nitzschia multistriata*, negatively affecting the growth of this and other diatom genera [21]. It colonises diatom cell surfaces, using their exudates to proliferate until diatoms reach the stationary growth phase. At this point, it leads to diatom death (likely to use the released organic matter), leaving the diatom cell surface before they sink. Similarly, *C. atlanticus* extracellular metabolites inhibit *T. pseudonana* cell division and alter its cell morphology, inducing an enlargement of the nucleus and an increased number of chloroplasts and cell size [104]. In addition, the expression of genes related to the biosynthesis and transport of carbohydrates, chrysolaminarin, and amino acids was increased in the diatoms supporting bacterial growth.

Also, vitamin B_12_-consumer bacteria can establish antagonistic interactions with diatoms, as in the case of *Methylophaga*, a gammaproteobacteria that competes for vitamin B_12_ while relying on phytoplankton carbon and energy to sustain its growth [87]. *Olleya* sp. A30, which possesses genes able to degrade diatom-derived organic compounds, seems be able to enter the frustules, consuming diatom organic matter from the inside [88]. When in co-culture with *P. subcurvata*, A30 negatively impacts the growth of this diatom by competing for vitamin B_12_.

Some bacterial genera affect, in a species-specific way, the efficiency of sexual reproduction [105]. *Maribacter* sp. and *Marinobacter* sp. cells and their spent medium, for example, negatively affect the sexual reproduction rate of the biofilm-forming pennate diatom *S. robusta*, generally more efficient in axenicity. On the other hand, *Roseovarius* sp. increased the proportion of sexually active *S. robusta* cells. In addition, *Maribacter* sp. and *Roseovarius* sp. both were able to modulate the levels of the pheromone diproline, which mediates the mate-finding process in diatoms [9], ultimately influencing the sexual reproduction in this species. In particular, *Maribacter* sp. affects the production of several amino acids and fatty acids indirectly correlated to diproline production, while *Roseovarius* sp. enhances the expression of enzymes responsible for the synthesis of diproline precursors [105]. Moreover, the *S. robusta* microbial community was able to interfere with the diatom’s pheromone system through the degradation of diproline, probably used as a carbon source in the absence of other sources, which indeed was more concentrated in axenic cultures.

Some marine proteobacteria frequently associated with diatoms use AHLs as signal molecules in QS [106]. In a biofilm matrix, AHLs can reach high local concentrations and be converted into tetramic acids (TAs). Using synthetic analogues, it was observed that both the N-(3-oxododecanoyl) homoserine lactone (OXO12) and its tetramic acid (TA12) inhibited the growth of the diatom *P. tricornutum*, with TA12 exerting this action at lower concentrations than OXO12. In addition, increasing concentrations of TA12, able to bind the quinone B (Q_B_) binding site in the photosystem II (PsII), caused a decrease in the diatom’s photosynthetic ability. On the other side, the benthic *Nitzschia* cf. *pellucida* seems to be able to disrupt bacterial β-keto-AHL signals using its haloperoxidase system to cleave its halogenated *N*-acyl chain [110]. Other than AHLs, other QS molecules, i.e., the quinolones, mediate diatom–bacteria interactions [107]. Quinolones are mainly produced by marine bacteria belonging to the *Pseudoalteromonas* and *Alteromonas* genera, but also by soil and freshwater bacteria belonging to the *Pseudomonas* and *Burkholderia* genera [107]. For some quinolones, the mechanism of action is still not understood, such as 2-pentyl-4-quinolone (PHQ), which inhibits the growth of *Cylindrotheca fusiformis* and *T. weissflogii* [108], and 2-n-pentyl-4-quinolinol (PQ), which impairs the motility of *Amphora coffeaeformis*, *Navicula* sp., and *Auricula* sp. when used to treat established biofilms, suggesting its use as a candidate antifouling agent [109]. On the other hand, a recent study highlighted the mechanism of action of 2-heptyl-4-quinolone (HHQ), which impaired *P. tricornutum* growth by inhibiting both photosynthetic electron transport and respiration [107].

#### 3.3.2. Algicidal Bacteria

Algicidal bacteria are important components in the determination of phytoplankton successions in marine environments since they can inhibit microalgal growth both through direct contact and the active release of diffusible factors to lyse cells [35,111]. The mode of action of algicidal bacteria ranges from high specificity for bacteria that kill only one species to universal for bacteria affecting a broad range of phytoplankton members [111]. For some bacteria, especially those living in biofilms, the algicidal activity is triggered by the perception of a certain microalgae density through a QS system [107], while others can switch on and off their algicidal activity depending on nutrient availability, with high nutrient concentrations stimulating the algicidal activity [112]. A simplified list of examples of algicidal bacteria and their effects on diatoms is reported in Table 4.

The most widespread algicidal bacteria belong to the genera *Saprospira*, *Vibrio*, *Alteromonas*, *Pseudomonas*, and *Pseudoalteromonas* [113]. Generally, *Alteromonas*, *Pseudoalteromonas*, and *Vibrio* kill diatoms, releasing diffusible substances, while other genera such as *Cytophaga* and *Flavobacterium* require direct contact with diatoms [34]. There are some exceptions reported, and one of them is *Kordia algicida*, a *Flavobacterium* that uses the QS-controlled release of diffusible proteases to kill a wide range of algal species, including the diatoms *S. costatum*, *T. weissflogii* [113], *C. socialis* [111], and *P. tricornutum* [34]. On the contrary, *Chaetoceros didymus* is not susceptible to *K. algicida* presence, and, surprisingly, the medium in which they are co-cultured is still active against *S. costatum* [34], a species susceptible also to the release of diffusible factors with protease and DNAse activities from other algicidal bacteria [114]. Another example of an attack mediated by diffusible factors is given by the bacterium *Pseudomonas* C55a-2 strain, which produces and releases 2,3-indolinedione to kill the diatom *Chaetoceros ceratosporum* [115].

An example of direct attack against diatoms by algicidal bacteria is represented by *Chitiniomonas prasina*, LY03 strain [116]. It employs chemotaxis to reach the *T. pseudonana* cells and chitinase to degrade the chitin present in the frustules, causing the loss of integrity of diatoms and the release of nutrients that are then used by the bacterium. Another bacterium directly attacking diatoms is *Cytophaga* sp. J18/M01, which targets different species of marine phytoplankton, including the diatoms *Ditylum brightwellii*, *C. didymum*, *S. costatum*, and *Thalassiosira* sp. [117]. Moreover, the bacterium *Saprospira* sp. strain SS98-5 uses its gliding motility, exerted through the production of microtubule-like structures in the bacterial cytosol, to reach the diatom *C. ceratosporum*, inducing diatom aggregation and subsequent cell lysis [118].

#### 3.3.3. Inhibitory Effects of Diatoms on Bacterial Growth

Some diatom species are able to activate defence mechanisms to deal with algicidal bacteria (simplified list of examples in Table 5) [4].

The inhibitory effects of diatoms on bacterial growth are often mediated by compounds that, in some cases, have been isolated and characterised. PUAs, for example, affect diatom-associated bacterial communities when present in high concentrations [45,123]. Pure 2E,4E-decadienal, 2E,4E-octadienal, and 2E,4E-heptadienal tested on 33 bacterial strains at unnaturally high concentrations showed a variety of effects ranging from concentration-dependent growth inhibition to no effect or growth stimulation [43,123]. However, strain-specific effects on bacterial strains isolated from the Mediterranean Sea were also observed in laboratory experiments using PUA concentrations similar to those found in nature. Indeed, bacteria belonging to Gammaproteobacteria were less affected compared to *Rhodobacteraceae*, whose abundance decreased by 21%, suggesting a role for PUAs in the regulation of diatom-associated microbiome composition [124]. Nevertheless, mesocosms studies on natural plankton communities enriched with the PUA-releasing diatom *S. marinoi* revealed no change in the shape and abundance of the bacterial community, suggesting that factors other than PUAs influence bacterial community diversity [43]. It cannot be ruled out that PUAs may have a role in affecting bacterial community growth and composition since they can reach very high local concentrations in biofilms or in TEP aggregates during blooms [45,123].

On the other side, oxylipins (oxygenated PUFA derivatives) are used by *C. didymus* against *K. algicida* attacks [34]. Indeed, differently from *S. costatum*, which is susceptible to the bacterial attack, *C. didymus* uses a wound-dependent defence mechanism to counteract the bacterial attack, which activates the synthesis and release of oxylipins [35], but also releases proteases [125]. The most abundant oxylipin released by *C. didymus* when attacked by *K. algicida* is the hydroxylated eicosapentaenoic acid 15-HEPE, which significantly inhibits bacterial growth [35].

*P. tricornutum* cell lysates showed antibacterial activity in vitro, which was attributed to the presence of eicosapentaenoic acid (EPA), palmitoleic acid (PA), and (6Z, 9Z, 12Z)-hexadecatrienoic acid (HTA), active against both marine and non-marine gram-positive and negative bacteria [120,121]. The analysis of the concentration of these fatty acids in the different *P. tricornutum* morphotypes highlighted higher production by fusiform cells with respect to the oval one [122]. Indeed, fusiform cells, which lack siliceous frustules and do not produce exopolysaccharides as protection, may invest more energy in fatty acid production to compensate for the absence of other defence mechanisms.

In some cases, even if the compounds have not been identified, diatoms show an inhibitory effect when co-cultured with target bacteria. As an example, in co-cultures of the benthic diatoms *Nitzschia laevis* and two strains of *Nitzschia frustulum*, *N. incerta*, *Navicula* cf. *incerta*, and *Navicula biskanterae* with *Vibrio alginolyticus*, *V. campbellii*, and *V. harveyi*, the diatoms were able to specifically inhibit the growth of these bacteria that are pathogenic for crustaceans, fish, and molluscs, which is a danger for aquaculture [119]. This study provides evidence for a possible “green” solution to control the growth of pathogenic bacteria by adding diatom species to culture water, thus avoiding the use of potentially dangerous chemicals in aquaculture plants.

The bactericidal activity of some diatoms, such as the one characterised by Desbois et al. 2008 and 2009 [120,121], can be further explored for biomedical applications. Indeed, PA and EPA from *P. tricornutum* are highly active against multidrug-resistant *Staphylococcus aureus* (MRSA), possibly finding applications towards drug-resistant bacterial infections [120,121]. There are also several examples, that are not directly involved in diatom–bacteria interactions, of diatom extracts with antibacterial activity, such as the ethanol extract of *S. costatum*, which showed growth inhibitory activity against *Streptococcus pyogenes* [126] and the crude extracts of *T. rotula*, which had high antimicrobial activity against *S. aureus*, *Bacillus pumilus*, and *Micrococcus luteus* and were also active against *Vibrio harveyi* [127]. This evidence suggests that marine diatoms can be further explored as a possible source of natural antibiotics.

## 4. Diatom-Associated Microbiomes

In the last few years, interest in studying the complexity of interactions established in nature between diatoms and their microbiome has increased, thus providing new information on their interplay [128], the stability of the associations and diatom species specificity [6], and the seasonality or geographical location influences [129]. As an example, a recent in situ study performed through metabarcoding of samples collected along the Australian coast in different seasons and locations allowed the characterization of the microbial community and reported that temperature and nutrient composition drive diatom community assemblages in different geographical locations. Moreover, they observed patterns of co-occurrence, conserved across space and time, among certain bacteria belonging to the *Roseobacter* and *Flavobacteria* clade with the diatoms of the genera *Skeletonema*, *Thalassiosira*, and *Cylindrotheca* [130], suggesting that species-specific interactions take place between these organisms and that those bacteria may significantly contribute to the seasonal and spatial variability of diatom communities.

In general, the structure of a diatom’s associated microbiomes may be determined, in some cases, by selective processes that are both environmental and host-guided, or in other cases, by neutral lottery-like processes, for which the possibility of a certain bacteria becoming part of a microbiome is proportional to its abundance in the environment [131]. In this context, Stock et al., [131] observed that the host-guided selection of the microbiome is predominant over the lottery-like one. Diatoms themselves indeed select microbiome members by providing nutritional conditions that favour the growth of certain species over the total community [23] and relying on different bacterial species/genera depending on the necessity to uptake nutrients that they are not able to produce, i.e., vitamins [84] and nitrogen [89], or to be protected from pathogenic bacteria and competing algal species [11]. Concurrently, associated bacteria benefit from algal excretions of DOM [8], whose quality and quantity represent one of the main driving forces shaping the microbiome composition [132].

Chemo-attractant and chemo-repellent compounds, but also secondary metabolites, released by diatoms in the phycosphere [4,8,16], can greatly influence the microbiome composition [11]. As an example, the analysis of the *P. tricornutum* exo-metabolome revealed the production of a pool of metabolites able to shape the associated microbiome composition, among which the 4-hydroxybenzoic acid selectively stimulated the growth of bacteria capable of metabolising and using it as a carbon source [133].

Axenic *A. glacialis*, when re-inoculated with its original microbiome, showed significant changes in its transcriptome and metabolome profiles and started secreting azelaic acid (AA) and rosmarinic acid (RA), able to favour the establishment of selected bacterial species while inhibiting the growth of unnecessary species [28].

On the other side, some bacteria species are able to control the composition of the microbiome associated with a certain diatom. This is the case of *Phaeobacter inhibens*, naturally occurring in association with *T. rotula*, which was shown to influence the microbiome assembly associated with the diatom [128]. Indeed, when axenic *T. rotula* cultures were exposed to natural seawater bacterial communities in the presence of *P. inhibens*, this bacterium led to a higher abundance of Alphaproteobacteria, Flavobacteriia, and Verrucomicrobia compared with cultures incubated only in the presence of natural bacterial communities.

Many of the cited studies have been conducted in laboratory conditions and revealed the long-term stability of the associated microbiomes, as for different strains of *Asterionellopsis glacialis* and *Nitzschia longissima*, which maintained a unique bacterial community for up to one year of growth in the laboratory [6]. In addition, species belonging to the *Roseobacter* clade were constantly present in all the analysed diatom strains and time periods. Also, the microbiomes of different *P. tricornutum* strains, despite belonging to different geographical locations, were very similar among each other and remained stable during laboratory cultivation, differing from the ones of other microalgal genera, i.e., *Entomoneis* and *Tetraselmis*, cultivated in the same conditions [134]. However, cultivation parameters conditioned the microbiome composition since, in the same *P. tricornutum* strains, it changed slightly when grown in a different medium with a higher nutrient concentration. Sison-Mangus et al. [102] showed that *Odontella* sp., isolated from a water sample in which the dominant bacteria were sex-promoting species belonging to the *Flavobacteriaceae* and *Rhodobacteraceae* families, maintained the association with them even after 130 generations in the laboratory. Barreto Filho et al. [135] also demonstrated that diatom microbiomes can remain stable during laboratory maintenance for approximately eight years in diverse diatom cultures. Di Costanzo et al. [103] reintroduced the original microbial community in an axenic *T. rotula* culture and followed for 8 months the presence of 3 newly identified bacterial species of particular interest for their biosynthetic potential, showing they remained in stable association with the diatom in this time frame. In another study, an axenic *T. rotula* exposed to bacterial inocula derived from different clones of the same species, congeneric species, and species belonging to different genera displayed a convergent and stable core microbiome, dominated by *Rhodobacteriaceae*, *Alteromonadaceae*, and *Oceanospirillares*, even after several months of culture [23]. Crenn et al. [136], analysing the epibiotic bacterial community, i.e., the bacteria attached to the diatom surface, of 436 *Thalassiosira* and 329 *Chaetoceros* cells isolated from both laboratory cultures and environmental samples, revealed a diverse and species-specific bacterial assemblage. In particular, the epibiotic bacteria in the laboratory cultures demonstrated to be less diversified than the ones in the environmental samples. In addition, the most frequently identified epibiotic bacterial species in the laboratory cultures were less abundant in the environment. Thus, the authors hypothesised that the uniformity of laboratory culture conditions may influence the establishment and maintenance of stable associations.

Some studies also showed that the microbiome composition of certain diatom species is greatly influenced by sampling time and location, which appeared to be more relevant than the species of diatoms considered [135]. As an example, the comparison between the microbiome compositions of *Cylindrotheca closterium* sampled in different marine habitats, although cultivated in the same laboratory environment, presented substantial variability [131]. A variation partitioning analysis conducted on *C. closterium* microbiomes and on randomised communities of bacteria sampled at the same sites where diatoms were collected demonstrated that sample location exerted the strongest influence on diatom microbiome composition, followed by host-related selection processes and the establishment of competitive interactions among associated bacteria. Moreover, host selection processes seem to favour the establishment of communities formed by a limited group of similar bacteria, thus reducing competition for common resources. It was also revealed that positive biotic interactions and a selection based on functional relationships increased the similarities between the bacterial communities of different *C. closterium* strains. In accordance with these results, Ajani et al. [129] demonstrated that 36 strains of three *Leptocylindrus* species sampled in six different locations during one year harboured bacterial communities whose composition was not significantly different when comparing diatom strains or species, being all dominated by *Rhodobacteraceae* and *Flavobacteriaceae*, but clearly different when comparing the time and location of sampling. On the contrary, other studies have highlighted that the host genotype has a greater influence than stochastic events in structuring the associated microbiomes [137]. Indeed, despite the analysis of the associated microbiome of 81 *T. rotula* genotypes isolated worldwide showing an absence of a core microbiome shared among all clones with a high degree of variability in their microbiome composition, a significant relationship between the host population and the taxonomic composition of its microbiome was found. The influence of the host genotype in shaping the microbiomes of this diatom was confirmed through a “common garden” experiment that showed highly reproducible bacterial communities associated with each genotype after 5 days of incubation with natural bacteria assemblages. In addition, closely related bacterial orders were found in association with different *T. rotula* clones, suggesting they may serve the same ecological role in the different environments from which the algae were isolated [137].

Only a few studies have been dedicated to the influence of the microbiome community on the diatom physiology. Baker and Kemp [138] showed that perturbations in diatoms’ microbiome composition caused by exposure to unfamiliar bacteria can affect diatom growth in the long term. In addition, abiotic stressors, such as vitamin availability, can modulate the effect of these perturbations. Indeed, bacteria belonging to the genera *Alteromonas* and *Marinobacter* re-inoculated in the *Chaetoceros* sp. strain KBDT20 (from which they were previously isolated), inoculated in the *Chaetoceros* sp. strain KBDT32, and in *Amphiprora* sp. contributed to the long-term effect on the growth and health status of these diatoms both under vitamin-replete and vitamin-deficient conditions. In particular, KBDT20 was unperturbed by the bacterial re-introduction in all the conditions tested; *Amphiprora* sp. was negatively impacted by the inoculation of *Marinobacter* only under vitamin-deficient conditions, while KBDT32 was impacted by both bacterial species depending on vitamin concentration: the impact on the growth rate was negative under vitamin-deficiency conditions and positive under vitamin-replete conditions.

## 5. Most Used Approaches to Study the Bacterial Communities’ Diversity

The technological advancement, especially in omics technologies, is greatly contributing to improving our understanding of microbial interactions, which are the subject of intensive investigations and projects based on the integration of big data coming from different approaches and omics techniques [139]. This approach integrates the analyses of meta-omics data to predict: (i) potential biotic interactions; (ii) reveal niche spaces; and (iii) guide more focused studies on possible relations and roles of compounds from different organisms composing the bacteria–diatom community. The co-occurrence pattern represents a useful tool to infer possible connections among microorganisms based on their abundances. An example related to the study of diatom–bacteria interactions in the marine environment derives from the analysis of the huge amount of data generated by the Tara Oceans expedition project, which allowed for the unveiling of the intricate interconnections between bacteria communities and their hosts [140]. These NGS-based metagenomics and meta-transcriptomic studies provided a snapshot of population composition and response to environmental stimuli, possibly inferring information on interaction dynamics in the communities, and, in some cases, the presence of new gene functions [140]. These approaches thus represent powerful methods to retrieve a global view on interactions established between diatoms and bacteria [1,140] and may allow, for example, to better understand how bacteria react to blooms of diatoms, or vice versa, how a community of diatoms or a single species responds to the presence of certain bacteria communities. At the same time, it may provide information on the phytoplankton community composition, including the identification of previously unknown species.

Another level of resolution is represented by the “trait-based” approach, which aims to reconstruct community biodiversity by tracking microorganisms’ functional traits, ultimately inferring ecosystem functioning [141]. In particular, this approach uses functional annotations to cluster genomes by functions that co-evolved and are possibly connected, which allows grouping both known and unknown microorganisms. The use of clustered functions simplifies the analysis of enormous metagenomics datasets and allows the simplification and unravelling of the intricate interconnected fluxes of functions at the base of complex communities [141,142].

Several bioinformatics tools have been developed to study microbial communities’ composition and functioning, of which a simplified list is reported in Table 6.

The bioinformatics pipeline IDBac is a useful tool to integrate information on bacteria, proteins, and secondary metabolite profiles obtained through MALDI-TOF MS analysis [143]. This approach is based on the assessment of bacterial traits, the identification of phylogenetic relationships, and the comparison of metabolic differences among hundreds of clones in a short time.

At a lower scale, a community made up of a single diatom species and its associated bacteria can be studied by a combination of cell sorting techniques and metagenome sequencing of all the species composing the microbial community. Sequence clustering allows them to associate a set of genomic sequences with each species composing the consortium. Subsequent phylogenetic analysis can lead to identification at a species or genus level [103,144]. More specifically, this pipeline includes the utilisation of the NCBI database BLASwares by the Geneious^®^ software [144] to identify and edit the 16S rDNA sequences found in the previous step and then the SILVA INcremental Aligner tool (SINA) to align the sequences [145]. ARB is also used to identify ribosomal sequences not identified with the other tools and steps [146]. Mothur [147] and NodeXL are useful tools to cluster the sequences based on their OTU and are used for network analysis and to visualise how communities are interconnected.

RAxML [148] is a tool used to generate a phylogenetic tree using GTRMIX as a rate distribution model and ECOLI to filter the sequences with the OTU in a defined position. The phylogenetic tree output of RAxML can then be used as input in UniFrac [149] to make comparisons among microbial communities using the phylogenetic information retrieved in the previous steps. PCoA analysis can then be added to analyse casual relationships.

Using software able to identify secondary metabolite gene clusters, it is possible to predictively characterise each species from a metabolic point of view. The most commonly used tools for this purpose are Prokka [150], which combines KBase (https://www.kbase.us/) and RAST [151] platform for rapid annotations, and PRISM [152] and antiSMASH [153], which provide the identification of biosynthetic gene clusters. This information can aid in deciphering the mechanism at the base of the persistence of certain bacteria species in the microbiome associated with specific diatoms. This clustered genomic sequencing approach is particularly helpful to characterise those bacteria species that are difficult to cultivate in the absence of the host or following isolation from the consortium [103].

Bioinformatics tools, allowing the mining and interconnection of enormous amounts of data, may be really helpful in the understanding of the diatom–bacteria community composition, biodiversity, and interactions. The software here reported provides useful information to characterise the diatom-microbiome community by allowing species identification, genome composition estimation in terms of the functions and secondary metabolite production, and the assessment of connections among the microorganisms based on shared functions in the community.

## 6. Potential Biotechnological Applications of Diatom–Bacteria Consortia

Bacteria have been considered for a long time as undesired contaminants in microalgae cultures grown for biotechnological purposes [154]. This view has now changed, and interactions between microalgae and their microbiome are being investigated for possible useful applications [155]. Nevertheless, most of the studies produced until now concern the use of consortia made of microalgal species in the *Chlorella*, *Scenedesmus*, *Tetraselmis*, and *Chlamydomonas* genera and their associated bacteria for the removal of nutrients from eutrophic waters, wastewater treatment, biofuel, and valuable compound production [154,156,157]. According to the available literature, fewer studies have focused on the specific use of marine diatom–bacteria consortia. A simplified list of the known or potential biotechnological applications of diatom–bacteria consortia is reported in Table 7.

The beneficial cooperative interactions between diatoms and bacteria may provide useful tools to optimise the productivity of microalgae cultivation, since in most cases they culminate in an increase in diatom growth and consequently in their biomass [68], as for *T. pseudonana*, which increased cell density by 35% when cultivated in the presence of *D. shibae* [67]. The stimulatory effect of bacteria on diatom growth finds application mainly in wastewater treatment, bioremediation [156,162], and biofuel production [154]. In this regard, there is an increasing number of studies driving the development of artificial consortia specific to each application based on the interaction mechanisms occurring in the consortia [157]. For example, excess organic nutrients contaminating waters can be assimilated by diatoms and bacteria, stimulating their growth and allowing the coupling of wastewater treatment with the cultivation of the microalgae for biomass production and its exploitation for the extraction of compounds of interest [162] or bioenergy [157].

The presence of bacteria may positively influence the tolerance and performance of diatoms cultivated in presence of pollutants [158]. Indeed, it has been observed that the co-occurrence of free-living bacteria belonging to the hydrocarbon-degrading genera *Marivita*, *Erythrobacter*, and *Alcaligenes* with the diatom *Nitzschia* sp. isolated from polycyclic aromatic hydrocarbons (PAHs)-contaminated sediments increased the growth rates and enhanced the tolerance of this microalga to PAHs compared to the performances of axenic diatom cultures, finally resulting in increased bioremediation of the PAHs fluoranthene (Flt) and benzo(a)pyrene (BaP) contaminated sites. This improvement in diatom tolerance to contaminants due to their associated bacteria has also been observed for *Thalassiosira delicatula* grown in the presence of metals and pesticide mixtures [163].

During the final steps of wastewater treatment, as well as in other industrial applications such as biofuel production, bacteria that increase microalgae flocculation through polysaccharides or protein production represents a sustainable, efficient, and cost-effective alternative to some toxic flocculation agents [155,162]. Nevertheless, the use of bacteria as flocculating agents in industrial applications is poorly explored for diatoms, but rather for other microalgae [164]. To the best of our knowledge, only chemical flocculation has been applied to *P. tricornutum* and self-flocculation to *S. marinoi,* as reviewed by Malik et al. [164]. A valid alternative to flocculation for biomass harvesting is also represented by the induction of biofilm formation, reducing the safety risks due to the use of toxic chemical flocculating agents [155]. This approach is also more advantageous compared to sedimentation and centrifugation, which are both time-, energy-, and money-consuming processes [19,165]. When grown in biofilms, consortia are able to remove a high quantity of nitrogen and phosphate from the wastewater, converting it into biomass [166].

Biofilms dominated by diatoms and bacteria can be also applied in aquaculture to improve larval fish growth [161]. Indeed, the biofilm forming naturally in *Seriola lalandi* culture cages, mainly composed of the diatom *N. phyllepta* and bacteria of the *Rhodobacteraceae* family, demonstrated the ability to control the proliferation of unwanted bacteria, especially when in combination with probiotic microorganisms, and to be a valuable nutritional source for the fish, being rich in carbohydrates. Biofilms can also be used as indicators of water quality because their composition and structure are susceptible to temperature increases and ocean acidification [65].

Bacteria may also assist diatom cell wall breakage to allow the release of their lipids and carbohydrates for biofuel production [159]. This can be achieved using algicidal bacteria that represent a cost-effective and environmentally friendly method to disrupt algal cells, since the compounds responsible for the effect are species-specific. Examples of promising bacteria that can be applied in biofuel production from diatoms are *Labrenzia* sp. KD531, which lyse *P. tricornutum* [159], considered a model system for biofuel production due to its high lipid content and easy cultivation, and *Marinobacter* sp., whose co-cultivation with this diatom increases its biomass and its total lipid content by 50% compared to axenic cultures [160].

Algicidal bacteria raised the interest of the scientific community also for their potential application in controlling harmful algal blooms (HABs), which can be detrimental for the environment, for aquaculture, and for human health due to the toxic effect of secondary metabolites produced by blooming microalgae [111]. The potential strategies that could be applied for the use of these “killer” bacteria in the control of HABs have been recently reviewed by Coyne et al. [167]. Despite the interest in using algicidal bacteria to control toxic diatom blooms, field and/or mesocosm experiments to assess the actual effect in marine environments are lacking.

Compared to applications related to bioremediation of polluted environments and improving water quality, only a few diatom–bacteria interactions have been exploited for pharmaceutical purposes. Indeed, the literature regarding the pharmaceutical, nutraceutical, and cosmetic applications of diatom–bacteria interactions is still scarce compared to the one regarding the same applications of diatoms and bacteria singularly. Vuong et al. [96] showed that production of fucoxanthin (a carotenoid with pharmaceutical, nutritional, and cosmetic applications) in *P. tricornutum* may be improved by *Stappia* sp. K01, which establishes a mutualistic relationship with the diatom, stimulating both diatom growth, which increased by 72%, and pigment production, which increased by 172%. Another example is given by the recently characterised sex-enhancing bacteria associated with *Odontella* sp. that could be exploited to increase the biomass and the long-term maintenance of this diatom and as a tool to genetically manipulate it in order to improve its health and cosmetic applications [102]. Of pharmaceutical interest could also be the proteases released in high amounts by *C. didymus* in the medium as a defence mechanism against the presence of the bacterium *K. algicida* [125]. Indeed, it has been reported that proteases can be exploited as possible tools for antiviral drug development [168].

Di Costanzo et al. [103], exploring the biosynthetic potential of bacteria associated with *T. rotula* and *S. marinoi* through the associated microbiome metagenome sequencing and the genome mining of three newly identified bacterial species strictly associated with the diatoms, showed the presence of novel hybrid clusters coding for non-ribosomal peptide synthase (NRPS)/Type 1 polyketide synthase (T1PKS), other than genes clustering for the biosynthesis of ectoine, terpenes, and bacteriocins. One of the three novel bacteria belonging to the *Roseivirga* genus also possesses 34 putative β-lactamase/cephalosporinases that can be exploited for the bioremediation of antibiotic-polluted waters.

Overall, there is still underexplored potential in the use of diatom–bacteria assemblages, which needs further efforts especially for marine species. This approach may, in the future, provide an improvement in the productivity yield of biomass or compounds of interest and more sustainable procedures to obtain products and services based on diatom cultivation.

## 7. Conclusions and Future Perspectives

The interactions between marine diatoms and bacteria can have a substantial influence on ecosystem functioning, as they affect primary production, phytoplankton aggregation, and carbon and nutrient fluxes. Although the relevance of this interplay is gaining increasing attention from the scientific community, more studies are needed for a deeper understanding of the mechanisms involved. Due to the complexity of the interactions occurring, new approaches and methods are being developed to reveal the interactome network and identify compounds responsible for chemical communication among the organisms. The increasing level of knowledge in this field will have a pivotal role in establishing new approaches to answer questions related to environmental changes, but also in finding new solutions for a sustainable exploitation of diatom-microbiome consortia for biotechnological applications, from bioremediation strategies to the development of valuable products for human wellbeing.

## Figures and Tables

**Table 1:** Examples of mutualistic interactions occurring between diatoms and bacteria, including the compounds involved and the resulting mutual behoof. Acronyms: NA = Not Available; DHPS = 2,3-dihydroxypropane-1-sulfonate; MMA = monomethyl amine; GBT = glycine betaine; Mas = methylamines; IAA = indole-3-acetic acid; DMSP = dimethyl sulfoniopropionate; FAs = fatty acids.

Bacterial Species	Diatom Species	Compounds Involved		Mutual Behoof	References
		Bacteria	Diatom		
*Ruegeria pomeroyi*	*Thalassiosira* *pseudonana*	Vitamin B_12_	DHPS	nutrients exchange	[85,86]
*Oceanospirillaceae* ASP10-02a	phytoplankton communities	Vitamin B_12_	Organic carbon	nutrients exchange	[87]
*Sulfitobacter* sp. SA1	*Pseudo-nitzschia* *subcurvata*	Biotin, thiamine, and vitamin B_12_	Organic carbon	nutrients exchange	[88]
*Richelia* *intracellularis*	*Rhizosolenia* and *Hemiaulus*	Fixed nitrogen	Amino acids and organic carbon	nutrients exchange	[89,90,91]
*Calothrix* *rhizosoleniae*	*Chaetoceros* spp.	Fixed nitrogen	Amino acids and organic carbon	nutrients exchange	[89,90,91]
KarMa strain of *Donghicola* sp.	*Phaeodactylum* *tricornutum*, *Amphora coffeaeformis* and *Thalassiosira pseudonana*	Ammonium	Organic carbon	Nutrient exchange based on the bacterial metabolization of environmental MMA	[80,92]
*Rhodobacteraceae*	*Phaeodactylum* *tricornutum*, *Amphora coffeaeformis* and *Thalassiosira pseudonana*	Ammonium	Organic carbon	Nutrient exchange based on the bacterial metabolization of MAs and GBT	[80,92]
*Sulfitobacter*, strain SA11	*Pseudo-nitzschia* *multiseries*	Ammonium	Organic carbon and DMSP	Nutrient exchanges based on IAA production by bacteria	[93,94,95]
*Stappia* sp. K01	*Phaeodactylum* *tricornutum*	NA	FAs	Nutrient exchange	[96]

**Table 2:** Examples of facilitative interactions occurring between diatoms and bacteria, including the compounds involved and the effects on diatoms. Acronyms: NA = Not Available; DKPs = diketopiperazines.

Bacterial Species	Diatom Species	Bacterial Compounds	Diatom Behoof	References
*Dinoroseobacter* *shibae*	*Thalassiosira* *pseudonana*	NA	increase in cell abundance and metabolic activity	[67]
*Bacillus thuringiensis*	*Phaeodactylum tricornutum*	DKPs	increase in growth and lipid content	[98]
Associated microbiome	*Halamphora* *coffeaeformis* and *Entomoneis paludosa*	NA	increased growth rates and cellular division but lower cellular amount of proteins, lipids and carbon	[99]
*Nautella* sp., *Sulfitobacter* sp., and *Polaribacter* sp.	*Chaetoceros* *tenuissimum*	NA	protection against the infection of the RNA virus CtenRNAV	[100]
Associated microbiome	*Thalassiosira* *weissflogii*/*Thalassiosira rotula*	Ectoine	survival to osmotic stress	[101,103]
*Polaribacter* sp. and *Cellulophaga* sp.	*Odontella* sp.	NA	increase in sexual cells and auxospores	[102]

**Table 3:** Examples of antagonistic interactions based on the inhibitory effects of bacteria on diatoms, including the compounds involved. Acronyms: NA = Not Available; OXO12 = N-(3-oxododecanoyl) homoserine lactone; TA12 = OXO12 tetramic acid; HHQ = 2-heptyl-4-quinolone; PHQ = 2-pentyl-4-quinolone; PQ = 2-n-pentyl-4-quinolinol.

Bacterial Species	Diatom Species	Bacterial Compounds	Effects on Diatoms	References
*Croceibacter atlanticus*	*Pseudo-nitzschia* *multistriata*	NA	induction of DNA fragmentation	[21]
*Methylophaga*	phytoplankton communities	NA	competition for vitamin B_12_	[87]
*Olleya* sp. A30	*Pseudo-nitzschia* *subcurvata*	NA	growth impairment	[88]
*Croceibacter atlanticus*	*Thalassiosira* *pseudonana*	extracellular metabolites	inhibition of cell division, alteration of cell morphology, increase in organic matter release	[104]
*Maribacter* sp. and *Marinobacter* sp.	*Seminavis robusta*	NA	negative influence on sexual reproduction rate by affecting diproline production	[9,105]
marine Proteobacteria	*Phaeodactylum* *tricornutum*	OXO12 and TA12	inhibition of growth	[106]
*Pseudoalteromonas* sp. and *Alteromonas* sp.	*Phaeodactylum* *tricornutum*	HHQ	Growth impairment by inhibition of photosynthetic electron transport and respiration	[107]
	*Thalassiosira weissflogii* and *Cylindrotheca* *fusiformis*	PHQ	inhibition of growth	[108]
	*Amphora coffeaeformis*, *Navicula* sp., and *Auricula* sp.	PQ	inhibition of motility	[109]

**Table 4:** Examples of antagonistic interactions, based on algicidal activity, occurring between diatoms and bacteria, including the compounds involved.

Bacterial Species	Diatom Species	Bacterial Mode of Action	Effects on Diatoms	References
*Kordia algicida*	*Skeletonema costatum*, *Thalassiosira* *weissflogii*, *Chaetoceros socialis*, and *Phaeodactylum tricornutum*	Release of diffusible proteases	death	[34,111,113]
*Pseudoalteromonas* sp.	*Chaetoceros didymus*, *Thalassiosira* sp., and *Eucampia zodiacs*	Release of diffusible factors with protease and DNase activities	death	[114]
*Pseudomonas*, C55a-2 strain	*Chaetoceros ceratosporum*	Release of 2,3-indolinedione	death	[115]
*Chitiniomonas prasina*, LY03 strain	*Thalassiosira pseudonana*	Release of chitinase	death	[116]
*Cytophaga* sp. J18/M01	*Ditylum brightwellii*, *Chaetoceros didymus*, *Skeletonema costatum*, and *Thalassiosira* sp.	direct contact with diatoms	death	[117]
*Saprospira* sp., SS98-5 strain	*Chaetoceros ceratosporum*	direct contact with diatoms	induction of aggregation and cells lysis	[118]

**Table 5:** Examples of antagonistic interaction based on diatom inhibition of bacterial growth and of the compounds involved. Acronyms: NA = Not Available; EPA = eicosapentaenoic acid; PA = palmitoleic acid; HTA = (6Z, 9Z, 12Z)-hexadecatrienoic acid.

Bacterial Species	Diatom Species	Diatoms Compounds	Effects on Bacteria	References
*Kordia algicida*	*Chaetoceros didymus*	15-HEPE	inhibition of growth	[35]
*Vibrio alginolyticus*, *V. campbellii*, and *V. harveyi*	*Nitzschia laevis*, two *Nitzschia frustulum* strains, *Navicula incerta*, *Navicula* cf. *incerta*, and *Navicula biskanterae*	NA	inhibition of growth	[119]
marine and not-marine gram-positive and negative bacteria	*Phaeodactylum tricornutum*	EPA, PA, and HTA	death	[120,121,122]

**Table 6:** Bioinformatic tools. Available software for bioinformatic analysis needed for the study of diatom bacteria interaction. Acronyms: NA = Not Available.

Software	Description	Website	References
IDBac	A MALDI Protein and Small Molecule Bioinformatics Platform	https://chasemc.github.io/IDBac/	[143]
Geneious^®^	Comprehensive suite of molecular biology and sequence analysis tools	https://www.geneious.com/	[144]
SILVA INcremental Aligner tool (SINA)	Web tool for multiple sequence alignment (MSA) specifically designed for the multiple alignment of ribosomal RNA genes (rRNA). SINA is also able to taxonomically classify the sequences.	https://bioinformaticshome.com/tools/msa/descriptions/SINA.html#gsc.tab=0	[145]
ARB	Graphically oriented package comprising various tools for sequence database handling and data analysis	http://www.arb-home.de/	[146]
Mothur	Single piece of open-source, expandable software to fill the bioinformatics needs of the microbial ecology community	https://mothur.org/	[147]
NodeXL	Makes it easy to explore, analyse, and visualize network graphs in Microsoft Office Excel	https://nodexl.com/	NA
RAxML	Popular maximum likelihood (ML) tree inference tool	https://raxml-ng.vital-it.ch/#/	[148]
UniFrac	Repository for high-performance phylogenetic diversity calculations repository for high-performance phylogenetic diversity calculations	https://pypi.org/project/unifrac/	[149]
Prokka	Software tool to annotate bacterial, archaeal and viral genomes quickly and produce standards-compliant output files	https://github.com/tseemann/prokka	[150]
RAST	Rapid Annotation using Subsystem Technology is a fully-automated service for annotating complete or nearly complete bacterial and archaeal genomes. It provides high quality genome annotations for these genomes across the whole phylogenetic tree.	https://rast.nmpdr.org/	[151]
PRISM	Combinatorial approach to chemical structure prediction for genetically encoded non-ribosomal peptides and type I and II polyketides	http://magarveylab.ca/prism/	[152]
antiSMASH	Tool for rapid genome-wide identification, annotation and analysis of secondary metabolite biosynthesis gene clusters in bacterial and fungal genomes. It integrates and cross-links with a large number of in silico secondary metabolite analysis tools that have been published earlier.	https://antismash.secondarymetabolites.org/#!/start	[153]

**Table 7:** Examples of diatom–bacteria consortia with their potential biotechnological applications. Acronyms: NRPS/T1PKS = non-ribosomal peptide synthase/Type 1 polyketide synthase; PAHs = polycyclic aromatic hydrocarbons.

Potential Biotechnological Applications	Diatom	Bacteria	Reference
Increase in biomass yield	*Thalassiosira pseudonana*	*Dinoroseobacter shibae*	[67]
Increase in pigments and biomass production	*Phaeodactylum tricornutum*	*Stappia* sp. K01	[96]
Increase in biomass yield	*Odontella* sp.	associated bacteria	[102]
Possible exploitation of NRPS/T1PKS, ectoine, bacteriocins, terpenes, and putative β-lactamase/cephalosporinases genes present in diatom-associated bacteria	*Thalassiosira rotula* and *Skeletonema marinoi*	Cluster 1, 2 and 8 bacteria	[103]
Increase in diatom-produced proteases by algicidal bacteria	*Chaetoceros didymus*	*Kordia algicida*	[125]
Bioremediation of PAHs in wastewater treatment	*Nitzschia* sp.	*Marivita*, *Erythrobacter,* and *Alcaligenes*	[158]
Diatoms disruption for biofuel production	*Phaeodactylum tricornutum*	*Labrenzia* sp. KD531	[159]
Increase in biomass and lipid yield	*Phaeodactylum tricornutum*	*Marinobacter* sp.	[160]
Increase in *Seriola lalandi* larval growth and viability in aquaculture	*Navicula phyllepta*	bacteria from the *Rhodobacteraceae* family	[161]

## Data Availability

No new data were created or analyzed in this study. Data sharing is not applicable to this article.
